# Training neural networks on domain randomized simulations for ultrasonic inspection

**DOI:** 10.12688/openreseurope.14358.1

**Published:** 2022-03-28

**Authors:** Klaus Schlachter, Kastor Felsner, Sebastian Zambal

**Affiliations:** 1 Machine Vision, Profactor GmbH, Steyr, 4407, Austria

**Keywords:** CFRP, Domain Randomization, Nondestructive Testing, Machine Learning, Defect Detection, Ultrasonic Rendering, Raytracing

## Abstract

To overcome the data scarcity problem of machine learning for nondestructive testing, data augmentation is a commonly used strategy. We propose a method to enable training of neural networks exclusively on simulated data. Simulations not only provide a scalable way to generate and access training data, but also make it possible to cover edge cases which rarely appear in the real world. However, simulating data acquired from complex nondestructive testing methods is still a challenging task. Due to necessary simplifications and a limited accuracy of parameter identification, statistical models trained solely on simulated data often generalize poorly to the real world. Some effort has been made in the field to adapt pre-trained classifiers with a small set of real world data. A different approach for bridging the reality gap is domain randomization which was recently very successfully applied in different fields of autonomous robotics. In this study, we apply this approach for ultrasonic testing of carbon-fiber-reinforced plastics. Phased array captures of virtual specimens are simulated by approximating sound propagation via ray tracing. In addition to a variation of the geometric model of the specimen and its defects, we vary simulation parameters. Results indicate that this approach allows a generalization to the real world without applying any domain adaptation. Further, the trained network distinguishes correctly between ghost artifacts and defects. Although this study is tailored towards evaluation of ultrasound phased array captures, the presented approach generalizes to other nondestructive testing methods.

## 1 Introduction

The field of applications of carbon-fiber-reinforced plastics (CFRP) expands with the progress in production of parts with complex geometries. However, the error-prone manufacturing process requires a seamless inspection of safety-critical components ^1^. For industrial production, this is typically performed by ultrasonic inspection. While the data acquisition process is often automated, the data still needs to be evaluated by trained experts. Such analyses are arduous and time intensive and lead to results dependent on the judgement of individuals. Thus, automated data evaluation has not only the potential of enormous time and cost savings, but also the potential to enhance the consistency of results.

Automated data evaluation in nondestructive testing (NDT) mostly relies on simple methods such as signal thresholding. For more advanced inspection cases, such as ultrasonic testing, inspectors utilize various signal characteristics. This complex decision does not offer a simple algorithmic description ^2^. Machine learning (ML) offers thereby an approach for automated evaluation devoid of manually derived algorithms.

A general problem for applying ML in NDT is the scarcity of flawed training data. For calibration and human training, artificially introduced defects, such as flat-bottom holes and side-drilled holes, are widely used. However, ultrasound signals of artificial flaws do not represent real data distributions ^3, 4^. While human inspectors can use their physical reasoning to judge real world data based on skills gained from training from simplified examples, training data for ML models need to represent the target domain.

Currently used strategies in NDT to overcome the data scarcity problem can mostly be grouped into data augmentation techniques and domain adaptation techniques. Data augmentation uses scan data from flawed samples. Extracting the flaw signals allows for the introduction of virtual flaws to arbitrary locations of the scan ^2^. Domain adaptation is a technique to adapt a model trained on a source domain (e.g. data from specimens with simplified artificial flaws like flat-bottom holes) to work on a target domain (e.g. real world data) ^5^. In the simplest case, this can be achieved by fine-tuning a pre-trained model with a smaller set of real world data.

Current approaches in NDT for utilizing simulations try to generate data as close to the real world as possible in order to get results which are useful in the real world or which at least allow the model to be adapted to the real world. The main idea of domain randomization is to introduce variations to the simulator itself. Enough variability might cause the model to see the real world just as another variation of the simulator ^6^. Recently, domain randomization was applied very successfully in various fields of autonomous robotics (e.g. for image-based pose estimation for robotic manipulation tasks ^7^, vision-based drone navigation ^8^, object category detection ^9^, and simulation-based robotic policy learning for autonomous quadrotor landing ^10^).

This article is structured in the following way. In Section 1.1 we review related work on ML applied in NDT. In Section 1.2 we provide an overview about simulating sound propagation in general. The derived phonon tracing algorithm for simulating total-focusing method (TFM) data is outlined in Section 2.1. The architecture and training of a neural network classifier is described in Section 2.2. An evaluation of the proposed approach and a discussion of results is provided in Section 3. Finally, an outlook is provided in Section 4.

### 1.1 Related work

In the recent years there has been considerable interest in developing machine learning models to evaluate ultrasonic data. Examples are monitoring of mixing processes ^11^, assessing carburization of industrial steel tubes ^12^, and testing of concrete foundation piles ^13^. We are particularly interested in applications regarding robot-guided ultrasonic testing of complex compact parts. ML could thereby enable (i) automatic defect detection, and (ii) adaptive path planning to guide the robot to areas of interest which require more detailed scanning ^5^. A major challenge towards the use of machine learning models for ultrasonic testing is the lack of representative training data. Data with real flaws tend to be scarce and producing mock-ups is expensive and time-consuming. Further, data is often confidential and therefore rarely shared, as it might reveal insights into the scanned structures ^2^.

**Data augmentation** has been suggested as a strategy to overcome the data scarcity problem in ultrasonic testing in the recent literature. The flaw signal is thereby extracted from a real scan and re-introduced at various positions. In 2 a machine learning model is trained with augmented data for flaw detection in phased array ultrasonic data. In order to increase the variety, various transformations, such as rotation and scaling, are applied on the extracted flaw signals. 

**Transfer learning** is another strategy applied in ultrasonic testing. Models pre-trained with data from another domain are thereby adopted to the target domain via transfer learning. In 14 a MobileNet is pre-trained on the ImageNet dataset (general image dataset) and migrated to feature extraction for a welding defect classificator. In 15 transfer learning is examined to monitor industrial processes via ultrasonic sensing. Two case studies on mixing and cleaning of fouled pipes are investigated and very promising results could be achieved ^16^.

**Training on simulated data.** Generating data via simulations provides many advantages to the methods mentioned above. The amount of data is easily scalable and accurate labels are accessible for free. Simulations also enable the integration of edge cases, which by definition rarely occur in the real world. However, statistical models trained in simulations often work poorly in the real world, and there is only very little literature examining this approach in the field of NDT. Impressive results were presented in 17 for defect detection in three-dimensional CT scans of cast aluminum parts. A deep segmentation model is trained solely on utilizing high-fidelity simulations. The segmentation model is able to deal with the huge variety of image artifacts of CT scans and achieves similar results as qualified experts. Further, it is shown that the model can be migrated to specialized tasks via fine-tuning.

**Domain randomization.** Generating realistic artificial data acquired from complex NDT methods is still a challenging task. Physical models need to make assumptions of the real world to keep the models manageable. More complex models enable reproduction of a broader variety of occurring physical effects, however, typically the number of parameters increases with the complexity of the model. To mimic the target system realistically, parameter identification can thereby be very challenging for more complex models. Domain randomization utilizes low fidelity data for training by introducing variability to the simulator itself. The main assumption is that with just enough variability in the simulator, the real world appears just as another variation ^6^. This approach is very successful in the field of autonomous robotics, however, there seems to be very little attention towards this technique in the NDT community.

**Ultrasonic image rendering.** Simulation of phased array ultrasonic data is used in various fields. In the NDT field simulations are used to evaluate influence factors and to optimize NDT configurations. To accurately account for heterogeneity and anisotropy, wave-based methods are utilized ^13, 18, 19^. In medicine, real-time rendering is of particular interest to enable medical training independent of availability of patients and to include rare diseases in the training ^20^. The simulator developed in this work is based on a geometric approach and mostly relies on efforts made in the medical field.

### 1.2 Simulation of sound propagation

Simulation of sound propagation is a widely studied field, motivated by a variety of use cases, e.g. auralization for audio post processing, auralization of virtual environments, and simulating acoustic properties of classrooms ^21– 23^. Lately, realistic ultrasound rendering is also studied to enhance medical training ^20, 24– 26^. Two main techniques have evolved for numerically evaluating sound propagation, with one group relying on a wave-based problem formulation, and the other relying on geometric acoustic approaches. The former approximates the solution of the underlying wave equation of linear acoustics for the pressure field *p*( ***x***, *t*)


$$\;\left(\frac{1}{c^{2}}\frac{\partial^{2}}{\partial t^{2}}-\nabla^{2}\right)p=0\;(1)$$

where *c* denotes the velocity at which the pressure disturbance propagates in the medium ^27^. The solution can be approximated by classical methods, e.g. by finite element method or boundary element method ^21, 28^. A review of the theoretical basis of these methods and their application for sound simulations can be found in 29. These methods provide very accurate solutions, but the computational costs are high and increase dramatically with the highest considered frequency. Hence, a wave-based approach is especially applicable for simulating low frequencies ^30^.

Geometric acoustics approximates sound propagation by sound particles moving along directed rays. This approach is suitable for waves with high frequencies, i.e. if the wavelengths are short in relation to the objects the wave is interacting with. Splitting the source signal into a low and a high frequency band allows combining geometric approaches with wave-based approaches in order to account for low frequency effects ^31^.

**Figure 1.**
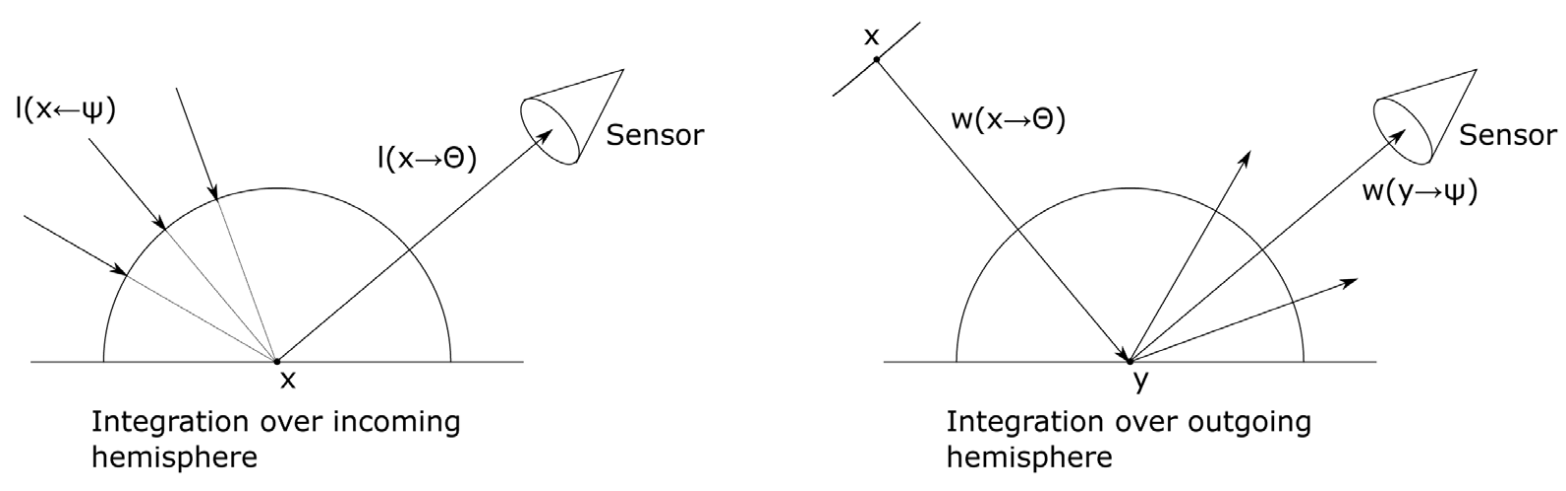
In the rendering equation integration is over the incoming hemisphere (left), whereas in the importance equation integration is over the outgoing hemisphere (right).

***1.2.1 The rendering equation*.** Approximating sound propagation via sound particles (phonons) along directed rays is very similar to the approach of geometric optics, and therefore many algorithms from light rendering can be adapted for sound simulations ^21, 31^. Light rendering is typically formulated as transport problem, where light sources emit energy which is transported in a three dimensional scene by means of reflection and refractions. The rendering equation, which describes this transport problem, was introduced to computer graphics by Kajiya ^32^. Thereby, it is assumed that light travels instantaneously and therefore steady-state is achieved instantaneously ^33^. In contrast, sound rendering has to account for the finite speed at which sound waves propagate in the medium. A thorough derivation of the acoustic rendering equation was presented in 34, which represents a time-dependent adaptation of the rendering equation. The rendering equation states that at a point *x*, the outgoing time-dependent radiance *l*( *x* → Θ) from a point *x* in direction Θ is the sum of the radiance *l _e_* ( *x* → Θ) emitted by the surface itself, and the reflected radiance *l _r_* ( *x* → Θ). As illustrated in Figure 1 on the left, in general, the reflected radiance results from partial contributions of incoming radiance l(x ←Ψ) from all directions Ψ. Integrating over the hemisphere Ω _x_, the (acoustic) rendering equation is stated as


$$l(x\to \text{Θ})=l_{e}(x\to \text{Θ})+\underset{l_{r}(x\to \text{Θ})}{\underset{︸}{\int_{\Omega_{x}}\rho (\text{Ψ},\text{Θ})l(x\leftarrow \text{Ψ})\cos(N_{x},\text{Ψ})\text{d}\omega_{\text{Ψ}}}}\;(2)$$

with the acoustic bidirectional reflectance distribution function (BRDF) *ρ* describing the acoustic reflection properties of the material. The cosine term accounts for geometrical foreshortening with the surface normal N _x_. 

***1.2.2 The importance equation*.** A rendering algorithm computes the light or sound energy that is visible at every pixel, while each pixel functions as a sensor. The importance equation - the dual of the rendering equation - was introduced to computer graphics by Pattanaik ^35^. The importance equation evaluates the contribution *w* of light or sound energy from point *x* in direction Θ to the sensor measurement. If the path ( *x*, Θ) reaches the sensor unhindered, the light or sound energy leaving *x* contributes fully to the measurement and therefore the so-called self-importance *w _e_* equals 1, otherwise it may reach the sensor through reflections and refractions and contributes partly. Given the BRDF *ρ* the importance equation is stated as


$$w(x\to \text{Θ})=w_{e}(x\to \text{Θ})+\int_{\Omega_{y}}\rho (\text{Ψ},\text{Θ})w(y\to \text{Ψ})\cos(N_{y},\text{Ψ})\text{d}\omega_{\text{Ψ}}.\;(3)$$

The rendering Equation 2 and importance Equation 3 describe the same transport problem; as both equations have the same mathematical structure, the same solution strategies can be applied. Ray tracing for solving the rendering equation sends rays from the sensor into the scene; backwards ray tracing (further referred to as phonon tracing) for solving the importance equation sends rays from the source into the scene ^33^.

## 2 Methods

In this section a phonon tracing algorithm which allows to generate artificial TFM data is derived. Subsequently, the architecture and training of a neural network for defect detection is described. The TFM simulator was implemented as Blender (v2.91) add-on and PyTorch (v1.9) was used for training.

### 2.1 Simulation of TFM images

TFM images result from post-processing of the captures of all transmitter-receiver pairs of a phased array. To emulate this, we simulate impulse responses by stochastic phonon tracing. The main emitting direction of a sender element is orthogonal to its surface, and the distribution of how the energy is spread around the normal is determined by the spatial dimensions of the sensor element with respect to the wave length. Array elements applied in non-destructive testing applications are typically rectangular. Further, the side along which the elements are arranged is typically significantly shorter than the width of the elements. This leads to a fan-shaped distribution of the emitted energy within the sensor plane.

**Figure 2.**
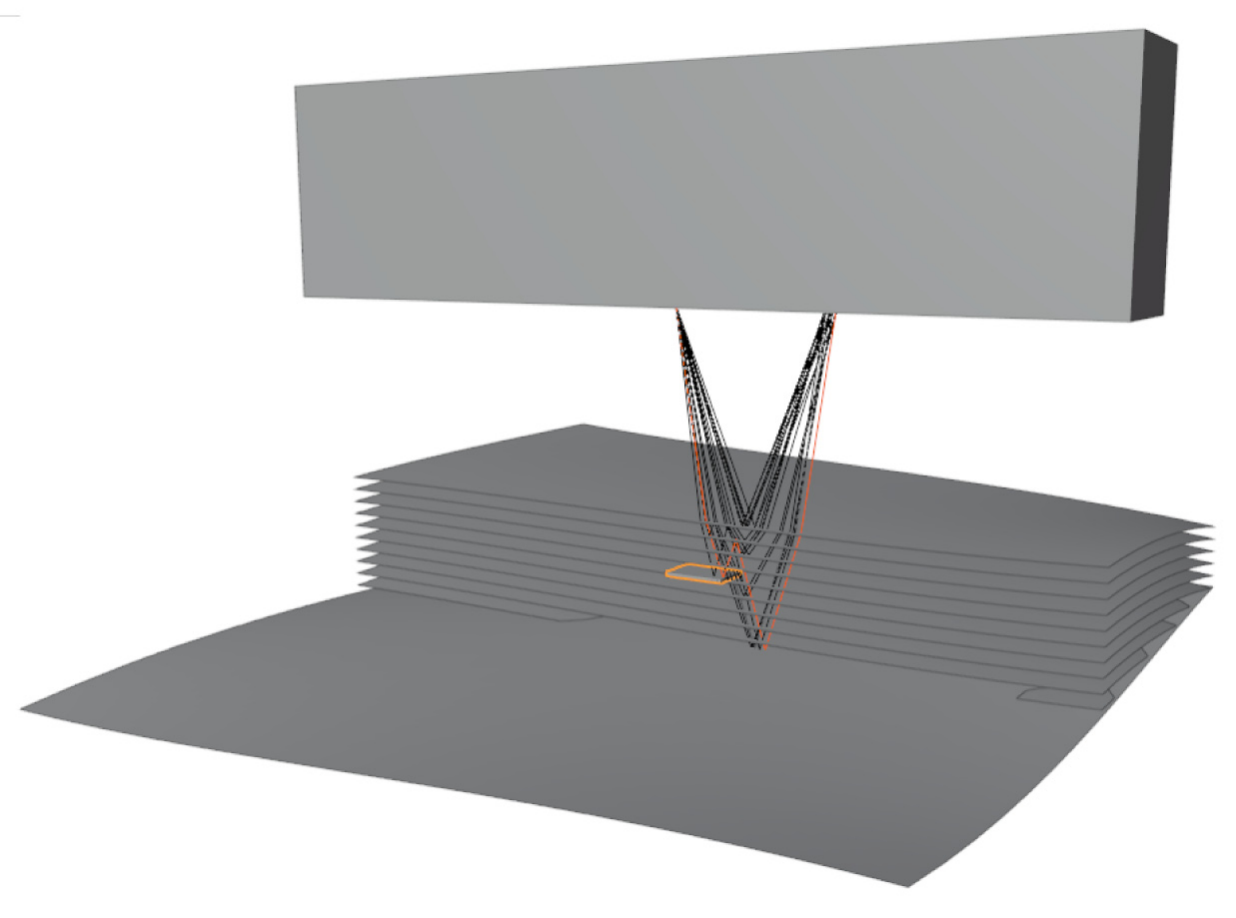
Sample showing a simulation of phased array sensor, artificial specimen, defect, and rays of a single sender-receiver pair.

An emitted phonon which hits a sensor element unhindered, contributes fully to the measurement at the time which the phonon took to travel from the emitter to the sensor. Otherwise, depending on the scene geometry, a phonon contributes to the impulse response through several reflections and refractions, and its contribution is determined by Equation 3. At the first interaction with a scene element at *y*, the energy is partly reflected and refracted. Additionally, the energy is spread out according to a distribution determined by the surface characteristics of the interface. Practically, these partial contributions are integrated numerically via Monte Carlo integration by sampling discrete directions Ψ _i_. Assuming that the self-importance equals zero, Monte Carlo integration of Equation 3, i.e.:


$$\;\hat{w}(x\to \text{Θ})=\frac{1}{N}\sum_{i=1}^{N}\frac{\rho \cos(N_{y},\text{Ψ})w(y\to {\text{Ψ}}_{i})}{p({\text{Ψ}}_{i})},\;(4)$$

calculates the importance estimation $\hat{w}$ . Thereby, the discrete directions Ψ _*i*_ are sampled from the distribution *p*. The importance values *w*( *y* → Ψ _*i*_) on the right-hand side of Equation 3 and Equation 4 are, however, unknown. Consequently, another evaluation is necessary. In general, the rays *y* → Ψ _*i*_ hit another interface at $y^{\prime }$ , resulting into nested integrals


$$w(x\to \text{Θ})=\int_{\Omega_{y}}\rho \int_{\Omega_{y^{\prime }}}\rho w(y^{\prime }\to \text{Ψ})\cos(N_{y^{\prime }},\text{Ψ})d\omega_{y^{\prime }}\cdot \cos(N_{y},\text{Ψ})d\omega_{y}\;(5)$$

with an increasing recursion depth for an increasing number of interfaces in the scene. In practice, these nested integrals can be solved by nested Monte Carlo integration schemes, leading to trees of paths traced through the scene. The number of nodes thereby increases exponentially with the number of intersections. Another strategy is to sample at every intersection a single direction in which the ray is further traced through the scene. As a result, more rays need to be sent into the scene. In this work each ray that hits a surface generates two follow-up rays – one accounting for reflection and one accounting for refraction. Figure 2 shows several rays emitted from an array element. These rays are traced through the scene, and received by another array element through several reflections and refractions. The simple

Monte Carlo integration scheme outlined above leads to an unbiased estimation, i.e. the expected value $\text{E}(\hat{w})$ equals the true value *w*. However, in practice the recursive evaluation requires a stop criterion. If a maximal recursion depth is introduced, paths are stopped via an upper bound for the number of echoes. Therefore, potential important signal paths are cut from the estimation, and a bias is introduced to the estimator. An alternative approach to this problem is known as Russian Roulette. At every interface, paths are stopped with some probability. This keeps the recursion depths manageable, however also a smaller number of long paths are produced. This allows for the exploration of the whole scene, with, in theory, paths with infinite recursion depth. To compensate for the lower probability of longer paths, each sample is weighted with the reciprocal of its continuation probability, which again results in an unbiased simulator ^33^.

**Figure 3.**
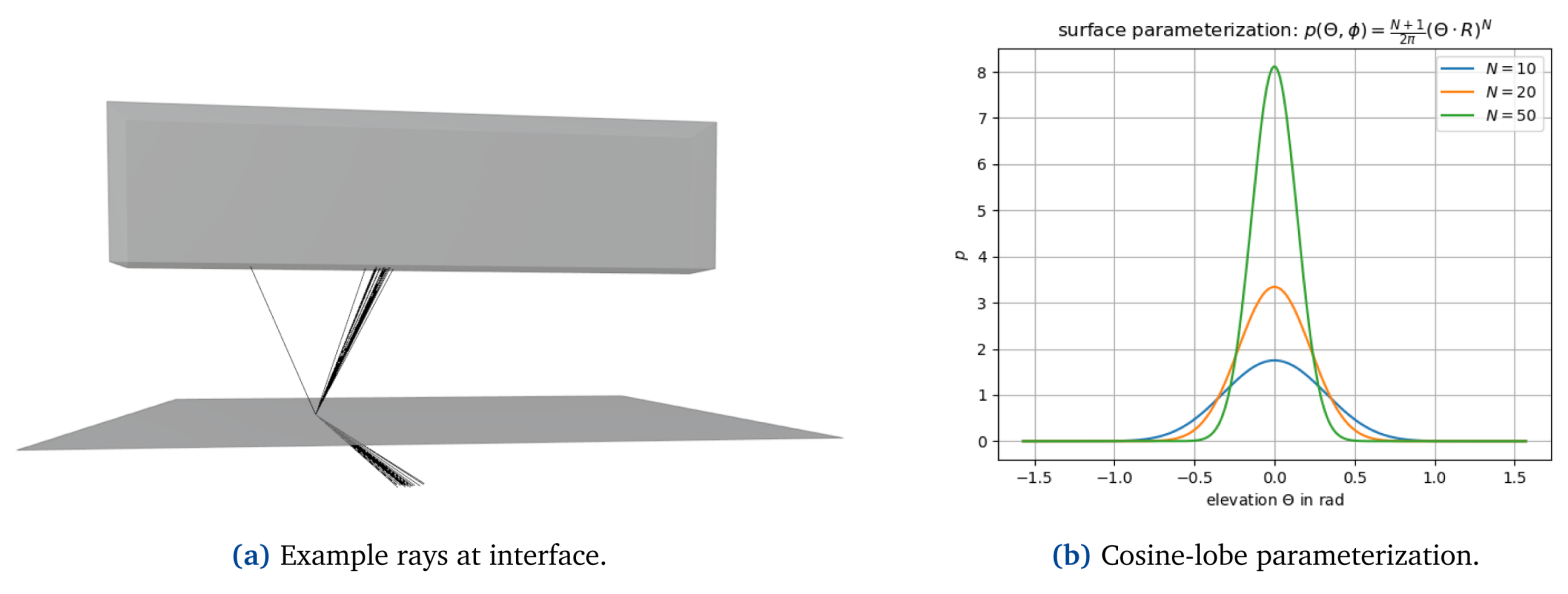
At interfaces, reflected and refracted energy is scattered according to a cosine-lobe surface parameterization ( **a**); ( **b**) shows the probability distribution used for sampling reflected and refracted rays with the elevation Θ, i.e. the angle between the ray and the specular reflection or refraction direction. The parameter *N* determines how strongly the energy is spread where *N* → ∞ representing the edge case of perfect reflection/refraction.


***2.1.1 Surface model*.** At a ray interface intersection, a reflected ray is generated with probability *p _R_* and a refracted ray is generated with probability *p _T_*, representing the fraction of energy which is reflected and transmitted. In order to account for the spreading of the reflected and refracted energy, we use a Phong-like cosine parameterization of the surface similar to 25. A ray is reflected in direction Θ according to the hemispherical distribution


$$\;\rho =k{\left(R\cdot \text{Θ}\right)}^{N}\;(6)$$

with the specular reflection direction R. The exponent N defines the shape of the function with the degenerated case N=0 for a perfect diffuse reflector and *N* → ∞ for a perfect specular reflector (see Figure 3). The normalization factor *k* depends on *N* and is chosen such that we obtain a valid distribution density function (integrating over the hemisphere must equal 1). The specular reflection direction R is calculated as

R=Θ−2(Θ⋅n)n.(7)

The direction of the refracted ray leaving a medium with refraction index *η*_1_ and entering a medium with refraction index *η*_2_ is calculated via Snell’s law


$$\;\eta_{1}\sin{\text{Θ}}_{1}=\eta_{2}\sin{\text{Θ}}_{2}\;(8)$$

with the angle of the entering ray Θ _1_ and the angle of the refracted ray Θ _1_ with respect to the surface normal.

In the context of Monte Carlo integration, in theory, every arbitrary probability function to generate samples produces an unbiased estimator. However, the variance of the estimator can be reduced by a suitable choice of the probability function (this is known as importance sampling). In practice, samples can be generated utilizing the surface model. This is obtained by sampling the azimuth angle uniformly in [0, 2 *π*]; elevation *R* · Θ is sampled by evaluating the inverse cumulative distribution *F* ^−1^( *u*) function at the uniform variable *u* ∈ [0, 1] where


$$\;\begin{array}{c} F(y)=1-\cos^{N+1}y\\ \;F^{-1}(u)=\arccos\;\sqrt[{N+1}]{1-u}. \end{array}\;(9)$$

As the cosine lobe is centered at the specular reflection (refraction) direction, there is a probability to sample a direction on the other side of the surface patch. In such a case, we simply resample a new direction.

***2.1.2 Implementation and image generation*.** For image generation, we shoot rays originated at the center of the transducer elements in directions sampled from a normal distribution. The intensity contribution to the final image is accumulated according to Equation 4. In order to sample reflected and refracted rays according to the described surface model, each surface in the scene is parameterized in terms of transmissivity, reflectivity, and the exponent *N* determining the cosine-surface parameterization in Equation 6. Each emitted phonon carries the following information:

- emitter index,
- receiver index,
- a weighting factor for Russian Roulette compensation and incorporating foreshortening,
- list of interface intersections to reconstruct the travelling time.

**Figure 4.**
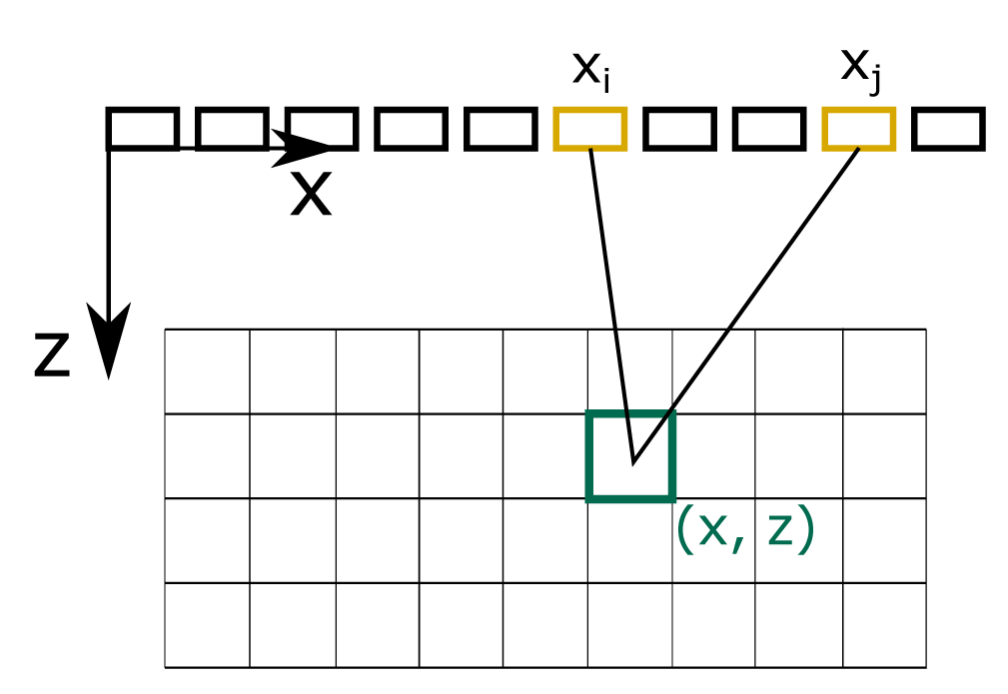
Total focusing method. Full matrix captures contribute to pixel intensity values according to path lengths from a sender *i* to a pixel at ( *x*, *z*) to a receiver *j*.

Algorithm 1 shows how the described strategies are combined. To explain how phonons contribute to individual pixels, we need to shortly introduce the TFM - the post-processing method of our target system. TFM was introduced in 36 and is one of the most popular methods for post-processing phased array data. The basis for image creation is a full matrix capture (FMC) of a phased array. This refers to a data acquisition that captures the responses of every possible transmitter-receiver combination. Figure 4 illustrates how the FMC captures contribute to pixel intensity values. First, the path length from the sender at ( *x _i_*, 0) to the pixel at ( *x*, *z*) to the receiver at ( *x _j_*, 0) is calculated. The Hilbert transform of the captured response function *h _ij_* – which is the envelope of the captured time signal – is evaluated at the calculated signal travelling time. Finally, the intensity value I of a pixel at (x, z) is calculated as the sum of the contributions of all sender-receiver pairs


$$I(x,z)=\sum_{i=1}^{N}\sum_{j=1}^{N}h_{ij}\left(\frac{\sqrt{{\left(x-x_{i}\right)}^{2}+z^{2}}+\sqrt{{\left(x-x_{j}\right)}^{2}+z^{2}}}{c}\right).\;(10)$$

**Algorithm 1. Stochastic phonon tracing**

**1 Input:** *x*, Θ, *w*, *max. recursion depth*

    
**if** *not reached max. recursion depth* **then**

      find next intersection *y* on *x* → Θ;

      
**if** *intersects with receiver* **then**

         add pixel contributions;

         return;

      Pick u uniformly from [0, 1);

      
**if** *u > nonTerminationProbabilty* **then**

         return;

      w *= nonTerminationProbabilty;

      Pick u uniformly from [0, 1)];

      
**if** *u < reflectivity* **then**

         sample reflected direction Ψ;

         w *= cos( *N _y_*, Ψ);

         trace reflected ray;

      Pick u uniformly from [0, 1);

      
**if** *u < transmissivity* **then**

         sample refracted direction Ψ;

         w *= cos( *N _y_*, Ψ);

         trace refracted vector;

To mimic this post-processing, we calculate the travelling time of a traced phonon, and evaluate to which pixels the phonon contributes according to Equation 10. Therefore, every phonon contributes to several pixels lying on an ellipse with foci being the centers of the receiver and transmitter element.

### 2.2 Domain randomized training

Simulating sound propagation in CFRP parts is a difficult task, as due to its composition its mechanical properties are highly anisotropic. The presented approach is a simple and easy to implement simulation which does not take into account more complicated wave phenomena, which arise due to a directional dependent wave velocity. Further, low frequency effects, such as diffraction, are ignored. This results into less rich, low-fidelity TFM images. However, these low-fidelity images contain some of our physical knowledge about the inspection system, i.e. reverberation, backwall attenuation, and reflections caused by delaminations. Therefore, some of our physical knowledge is exposed to the classifier during training. The hypothesis of domain randomization is that if the variability in simulation during training is sufficiently large, the trained model will generalize to the real world without additional training ^6^. Furthermore, by providing a wide range of (unrealistic) data, but which still contains important physical phenomena, that are important to judge the integrity of a specimen, some knowledge can be transferred to and therefore exploited by the ML model.

**Figure 5.**
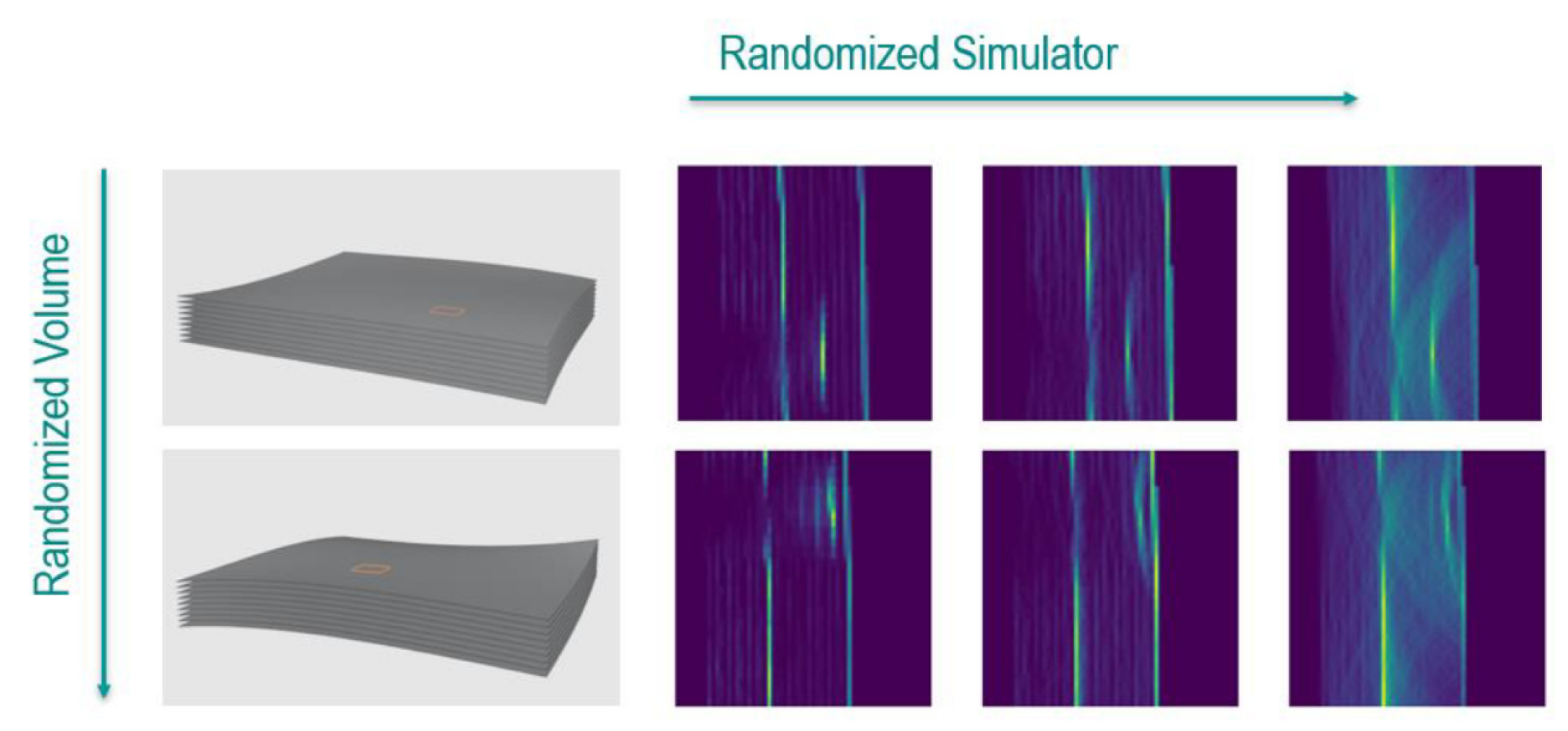
Randomization for artificial ultrasound data generation. The shape of the specimen and the location and size of the defect is randomized (columns). Artificial total-focusing method (TFM) data is simulated with randomized values for transmissivity and reflectivity (row).

**Parameter identification.** For simulating TFM images with our simulator as outlined in the previous chapter, parameter identification is required. Besides the TFM post-processing the considered inspection system also applies a depth-dependent gain to compensate for signal attenuation. To generate training data, we simply adjust the interface parameters by hand in order to achieve a similar outcome compared to our reference system. In particular, we tune the parameters in such a way that we get similar data without applying any post-processing. These values are used as mean values for later parameter randomization. For this purpose, we set the transmissivity of the CFRP sheets to 1 and adjusted the reflectivity in such a way that we get a similar outcome to the reference TFM images. In the same way, as a depth dependent gain corrupts the physical interpretation of received signal energies, the sum of reflected and refracted energy due to this choice is higher than the incident energy. Figure 5 shows examples of the used training data. The shape of the specimen is defined via nurbs surfaces, which are randomized. These randomized shapes are scanned with the simulator with randomized values for transmissivity and reflectivity.

**Figure 6.**
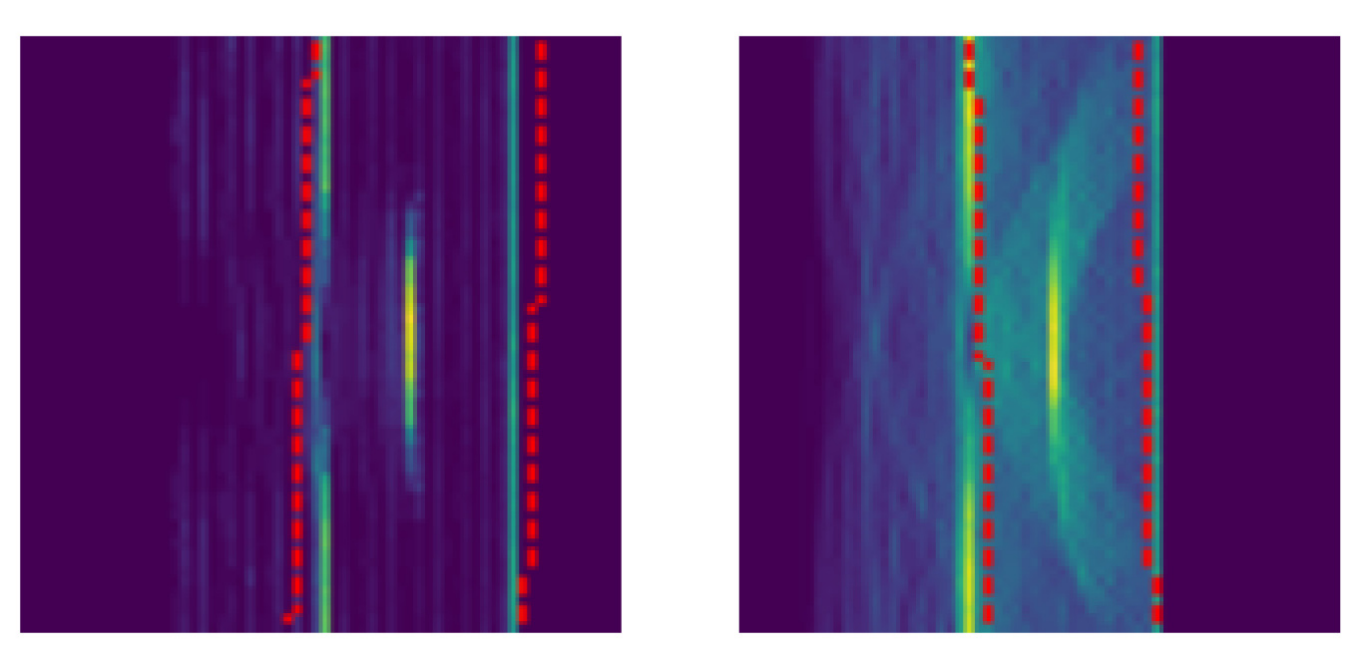
Training was conducted with volume masks as additional input. To disturb the masks, random rotation from -3 to 3 degrees and a random scale from 0.9 to 1.1 were applied. The resultant volume masks are indicated by the red-dotted line.

***2.2.1 Model architecture and training*.** For semantic segmentation we use a convolutional neural network with a U-Net architecture. This architecture - originally proposed for segmentation of medical volume data by Ronneberger, *et al*. in 37 - proved itself to be very successful in a variety of use cases. The network parameters are initialized randomly. Adam optimization is used to minimize the negative log likelihood loss function with mean reduction. As the ideal shape of inspected specimens is typically known, this information is often cheap and easy to integrate for evaluation. Therefore, we decided to additionally provide a distorted version of the volume as input. Distortions are achieved by applying random rotations in the range of [-3°, 3°] and by scaling the object with a factor from [0.9 to 1.1]. Examples of distorted input volume masks are shown in Figure 6. For rendering TFM images according to Algorithm 1 we sent 150 rays per element into the scene. The non-termination probability was set to 0.95 and the maximum recursion depth was set to 30.

## 3 Results and discussion

**Figure 7.**
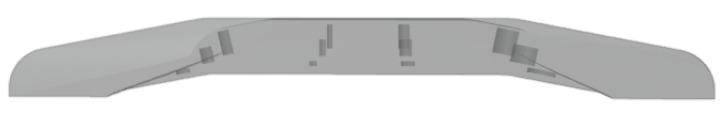
Flat-bottom holes introduced into reference specimen.

To verify the approach, we used scans gathered from a CFRP reference specimen, illustrated in Figure 7. Flat-bottom holes were introduced into the part as artificial reference defects (reflectors). This is a common practice for developing and verifying ultrasonic NDT methods. In total, the sample contains twelve flat-bottom holes, which are placed in the following pattern: four defects with varying radii are introduced within a single plane. Radii from left to right measure: 3 mm, 1.5 mm, 3 mm, 6 mm. This is repeated three times, while in each subsequent plane the defects are introduced in different depths, resulting in four defects with a distance of 1mm from the backwall, in the middle of the part, and with a distance of 1 mm from the frontwall. The reflectors (i.e. flat-bottoms of holes) are parallel to the frontwall of the specimen. The front and backwall of the specimen are slightly tilted resulting in different thicknesses in each plane, ranging from 7 mm to 10 mm.

**Figure 8.**
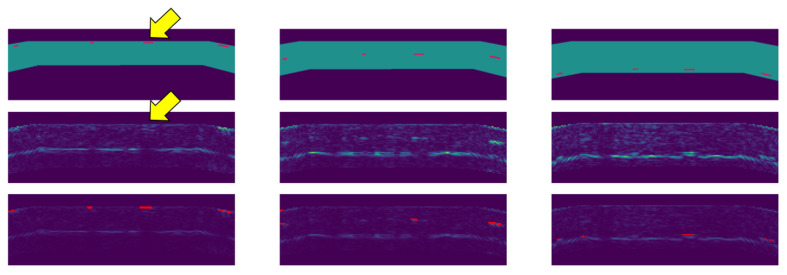
Defect detection on the reference specimen. The individual columns show section images at the location of flat-bottom holes close to the top surface (left), in the middle of the part (center), and close to the backwall (right). The top images show the part geometry and locations of flat-bottom holes. The middle images show original images with full brightness. The bottom images show the original data with reduced brightness and detections overlaid in red. The echo of a defect (yellow arrow ^1^) is correctly classified as background.

The ultrasound sensor used for scanning the reference specimen is a 5MHz linear phased array that consists of 114 transducers and has a width of 114 mm. The TFM images have a resolution of 0.1 mm and 16 bit depth. When feeding real data to the machine learning model, it outputs reasonable results. An example is shown in Figure 8. Thereby we provided volume masks obtained from a digital model of the specimen.

**Figure 9.**
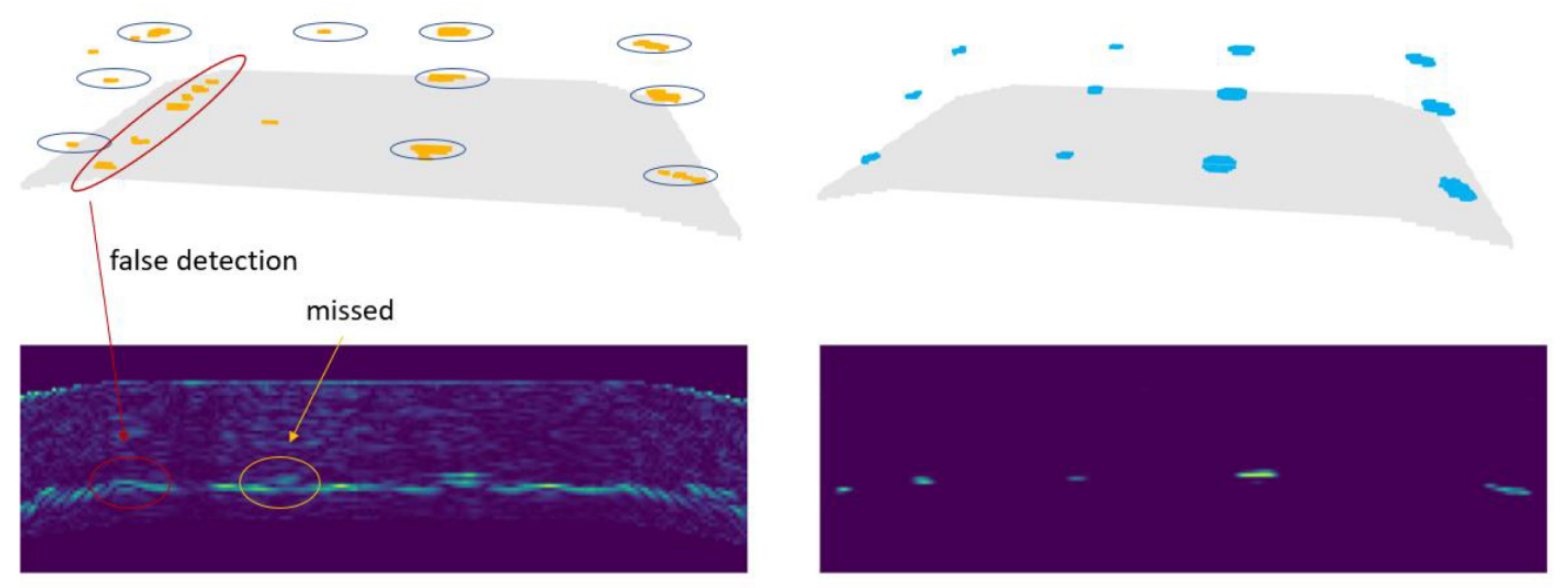
The top row shows the defect detection result (left) and the ground truth data (right) as 3d point clouds. The backwall of the specimen is indicated by a gray surface. The bottom row shows the input image (left) and defect probability output of the U-Net (right) of a single slice. ^2^

Due to limited availability of scan data, the evaluation of defect detection on a more quantitative level was difficult. Nevertheless, the developed defect detection method seems very promising. An interesting detail is that the method correctly classifies defects near the frontwall. Furthermore, the echo from a defect close to the top surface is correctly classified as background, i.e. as “no defect” (yellow arrow in Figure 8). The top row in Figure 9 shows the result of defect detection as 3d point cloud, the backwall of the specimen is indicated by a gray surface. Yellow points show the resulting output of the defect detection, blue points on the right show the locations of flat-bottom holes (voxelized ground truth).

In Figure 9, defects in the left two rows are those with 3mm and 1.5mm diameter. The two rows on the right represent defects with 3mm and 6mm diameter. The larger defects were detected by the trained neural network. Concerning the 1.5 mm diameter defects, the model failed in finding one of them in the middle and one closer to the backwall. Further, false-positives occurred at the transition between the inclined and the horizontal section of the backwall.

## 4 Conclusion

A simulator with low computational costs for rendering artificial TFM images is introduced. This simulator generates low-fidelity images, which yet reproduce important physical effects and artifacts. Domain randomized training of a semantic segmentation model was conducted without any domain adoption techniques. Thereby, in addition to the TFM images, distorted volume masks are fed into the network. The network, which was trained solely on artificial data, achieves reasonable results when applied to real world data. Further, the network correctly differentiates between real artifacts and ghost artifacts, which is known as a hard problem.

Domain randomization could achieve impressive results in various fields, however its potential is yet not much investigated in the NDT community. Results are promising, and we aim at a wider evaluation on real data from industry in the future. Training on simulated data could ease training for specialized cases where data is scarce and data of edge cases are not available. Besides a training solely on artificial data, the proposed method could serve as pre-training step and enable fine-tuning with small real world data sets.

False-positives occur almost exclusively on sharp corners of the specimen. This could be caused by the used specimen model, which is very smooth. In future work, a geometrically richer virtual specimen will be used, which better represents the considered use case.

## Data availability

### Underlying data

The reference specimen used in this work is owned by Fokker Landing Gear and the National Aerospace Laboratory (NLR). The authors were given access to the specimen as part of the Clean Sky 2 project under grand agreement No 831830. The raw ultrasonic dataset is held under restriction to internal use only, as a third party with access to the raw ultrasonic data may be able to reverse engineer very specific details about material type and laminate layup, which may compromise the GKN Fokker commercial position. In general, data with real flaws tend to be scarce, and data is often confidential and rarely shared. Due to the lack of existence, representative open source data cannot be provided.

## Ethics and consent

Ethics approval and consent were not required for this study.

## Footnotes

^1^Annotations were added in Powerpoint.

^2^3d point clouds were visualised with open3d in python and annotations were added via Powerpoint.
