# Supplementary material for: Training neural networks on domain randomized simulations for ultrasonic inspection
Source: Open Res Eur. 2022 May 11;2:43. Originally published 2022 Mar 28. [Version 2] doi: 10.12688/openreseurope.14358.2 (PMC10446096; doi:10.12688/openreseurope.14358.2)
Supplement: Peer review history

# Peer review: Reviewer response for version 2

**DOI:** 10.21956/openreseurope.15998.r29193

**Published:** 2022-05-23

**Authors:** Iikka Virkkunen

**Affiliations:** 1 Aalto University, Espoo, Finland

The authors address an important topic, i.e. the various ways to handle data scarcity in using machine learning techniques for NDE data evaluation. In particular, the authors target defect detection in TFM data frames acquired from carbon fibre composite components using U-net style segmentation network. The main contribution is to train the network using low fidelity simulated responses with the hypothesis that even substantially limited simulation based training data will provide sufficient generalization for finding defects, provided that the training data is sufficiently varied. 

 As a previous reviewer has already pointed out, there's wide variety of available methodologies for simulating ultrasonic responses, and the selection here is by no means obvious. Thus, a better justification of the chosen modelling approach would be warranted. Perhaps the choice was made in favor of a fairly simplistic modeling approach in order to study the value of even limited fidelity training material. 

 The results from the ML segmentation are compared against flat-bottom holes of various sizes. The authors state, that these kind of simplified flaws are commonly used develop and verify ultrasonic NDT methods. This is correct as far as providing reference signals for human inspectors go. However, using such simplified flaws to assess quality of ML training requires more justification. In particular here, where the object of study is the value extracted from simplified training data, comparing with simplified reference flaws may yield overly optimistic results. To balance, the authors do not claim high performance, instead, the performance is described as "promising" or "reasonable". Nevertheless, some discussion on the limitations of the current approach would be warranted. In addition, it would be good to give some sort of reference to the reader on the difficulty of these flaws: would a human inspector be expected to find all of them easily? Would the missed defects be considered difficult for the human inspector?  

 The above potential improvements notwithstanding, the paper presents a clear contribution to the field and suggests an additional tool to aid training state of the art machine learning networks with limited NDE data.

Is the rationale for developing the new method (or application) clearly explained?

Yes

Is the description of the method technically sound?

Yes

Are the conclusions about the method and its performance adequately supported by the findings presented in the article?

Yes

If any results are presented, are all the source data underlying the results available to ensure full reproducibility?

Partly

Are sufficient details provided to allow replication of the method development and its use by others?

Yes

Reviewer Expertise:

NDE reliability, Machine learning for NDE

I confirm that I have read this submission and believe that I have an appropriate level of expertise to confirm that it is of an acceptable scientific standard.

# Peer review: Reviewer response for version 2

**DOI:** 10.21956/openreseurope.15998.r29253

**Published:** 2022-05-12

**Authors:** Carmelo Mineo

**Affiliations:** 1 University of Palermo, Palermo, Italy

The authors have addressed all my comments appropriately.

Is the rationale for developing the new method (or application) clearly explained?

Yes

Is the description of the method technically sound?

Yes

Are the conclusions about the method and its performance adequately supported by the findings presented in the article?

Partly

If any results are presented, are all the source data underlying the results available to ensure full reproducibility?

Partly

Are sufficient details provided to allow replication of the method development and its use by others?

Partly

Reviewer Expertise:

Non-destructive testing, Ultrasonics, Ray Tracing, Phased Array, Composite materials, Robotic and autonomous inspections

I confirm that I have read this submission and believe that I have an appropriate level of expertise to confirm that it is of an acceptable scientific standard.

# Peer review: Reviewer response for version 1

**DOI:** 10.21956/openreseurope.15495.r28936

**Published:** 2022-04-11

**Authors:** Carmelo Mineo

**Affiliations:** 1 University of Palermo, Palermo, Italy

The authors present an interesting work to overcome the data scarcity problem of machine learning for non-destructive testing. They propose a method to enable training of neural networks on simulated data. This is aligned with my research interests and I appreciated the invitation to review this manuscript. The article is well written. The background motivations are clear. I think this work contributes to the state-of-the-art related to automated flaw detection in ultrasonic non-destructive testing. The authors should address the following questions/issues:

 About ultrasonic ray-tracing, the authors state: “The solution can be approximated by classical methods, e.g. by finite element method or boundary element method ^21,28^.” The authors seem to not be aware of recent solutions that have been proposed to solve the ultrasonic ray-tracing problem using iterative root-finding methods. These methods are described in the following publications:

1. Mineo, C., Lines, D., & Cerniglia, D. (2021). Generalised bisection method for optimum ultrasonic ray tracing and focusing in multi-layered structures. Ultrasonics, 111, 106330.
2. Mineo, C., Cerniglia, D., & Mohseni, E. (2022). Solving ultrasonic ray tracing in parts with multiple material layers through Root-Finding methods. Ultrasonics, 124, 106747.

The authors should explain the advantages offered by their ray-tracing solution. The description of the approach used for the simulation of sound propagation occupies most part of the manuscript. In my opinion, the authors should explain why existing approaches were not suitable to support their work on neural network training. In section 2.2, the authors state: “The presented approach is a simple and easy to implement simulation which does not take into account more complicated wave phenomena, which arise due to a directional dependent wave velocity.”. It is not clear why the authors have not tried to use existing and more accurate solutions. Was that deemed unnecessary? If that is the case, why?

 The ray tracing approach proposed by the authors seems to be capable of dealing with complex geometries. The authors say they have randomized the part geometries by applying random rotations in the range of [-3°, 3°] and by scaling the object with a factor from [0.9 to 1.1]. It is not clear if these ranges were limited by the capability of the ray-tracing approach to work with excessive object curvature of if they were randomly chosen by the authors. If the latter is the case, what is the maximum object curvature the approach can tolerate?

 In the text following Equation 8, replace “refracted ray Θ _1_” with “refracted ray Θ _2_”.

 In section 3, the following statement seems to contain a contradiction: “An interesting detail is that the method correctly classifies defects near the frontwall. Furthermore, the echo from a defect close to the top surface is correctly classified as background, i.e. as “no defect” (yellow arrow in Figure 8).”. I think the two sentences in the text above refer to the same subject, but have the opposite meaning.

Is the rationale for developing the new method (or application) clearly explained?

Yes

Is the description of the method technically sound?

Yes

Are the conclusions about the method and its performance adequately supported by the findings presented in the article?

Partly

If any results are presented, are all the source data underlying the results available to ensure full reproducibility?

Partly

Are sufficient details provided to allow replication of the method development and its use by others?

Partly

Reviewer Expertise:

Non-destructive testing, Ultrasonics, Ray Tracing, Phased Array, Composite materials, Robotic and autonomous inspections

I confirm that I have read this submission and believe that I have an appropriate level of expertise to confirm that it is of an acceptable scientific standard, however I have significant reservations, as outlined above.

## References

[1] : Generalised bisection method for optimum ultrasonic ray tracing and focusing in multi-layered structures. *Ultrasonics* .2021;111: 10.1016/j.ultras.2020.106330 106330 10.1016/j.ultras.2020.106330 33340820

[2] : Solving ultrasonic ray tracing in parts with multiple material layers through Root-Finding methods. *Ultrasonics* .2022;124: 10.1016/j.ultras.2022.106747 10.1016/j.ultras.2022.106747 35405599

# Response

**Published:** 2022-05-09

**Authors:** Klaus Schlachter

**Affiliations:** Profactor GmbH, Austria

Dear Carmelo Mineo, Thank you very much for your constructive review of our work. We appreciate your comments and suggestions to improve the manuscript. Please find our answers to the points raised and comments below. Our responses are shown in italics:

- About ultrasonic ray-tracing, the authors state: “The solution can be approximated by classical methods, e.g. by finite element method or boundary element method ^21,28^.” The authors seem to not be aware of recent solutions that have been proposed to solve the ultrasonic ray-tracing problem using iterative root-finding methods. These methods are described in the following publications:

1. Mineo, C., Lines, D., & Cerniglia, D. (2021). Generalised bisection method for optimum ultrasonic ray tracing and focusing in multi-layered structures. Ultrasonics, 111, 106330.
2. Mineo, C., Cerniglia, D., & Mohseni, E. (2022). Solving ultrasonic ray tracing in parts with multiple material layers through Root-Finding methods. Ultrasonics, 124, 106747.

*Thank you very much for pointing this out. Indeed, the introduction section regarding geometric acoustics was rather general, and we think it benefits from including more specific research that applies geometric acoustics for solving problems related to ultrasonic testing. We have added the following paragraph to the manuscript:*

*“In general, paths between two points are determined by an underlying variation problem known as Fermat's principle. In homogenous media rays travel along straight lines and change directions at boundaries of media with different wave velocities. Modern root finding methods allow to compute sound paths between a source and target point in multi-layered structures, such as composite parts, very efficiently [Mineo2021, Mineo2022]. For this purpose, the problem is modeled based on Snell's law. This approach can be extended for determining sound paths in media with continuously changing material properties by discretizing the media through regions with constant wave velocities. Such an approach can be applied to various problems in the NDT field, e.g. for efficiently determining ultrasonic paths for in-process inspections of welds at the presence of high temperature gradients. While such problems can also be tackled by finite element models, such an approach is of great advantage in terms of the required simulation time [Mineo2021, Mineo2022].”*

- The ray tracing approach proposed by the authors seems to be capable of dealing with complex geometries. The authors say they have randomized the part geometries by applying random rotations in the range of [-3°, 3°] and by scaling the object with a factor from [0.9 to 1.1]. It is not clear if these ranges were limited by the capability of the ray-tracing approach to work with excessive object curvature or if they were randomly chosen by the authors. If the latter is the case, what is the maximum object curvature the approach can tolerate?

*As shown in Figure 6 and described in subchapter “Model architecture and training” we provide a volume mask as an additional input channel to the segmentation model because the ideal shape of an inspected specimen is typically know. However, due to manufacturing tolerances, mesh registration inaccuracies (assuming an automated robotic inspection system), and limited robot trajectory accuracy, it is not reasonable to assume that captured TFM images are perfectly aligned with production drawings. Therefore, this additional input channel is meant to provide context information, however, in order to prevent the neural network from simply relying on the volume mask, the volume mask was distorted within a reasonable range.*

*Indeed, the virtual model used for training is pretty smooth, though, we do not see any obvious reason why the presented approach should be limited by curvature. We think that the training would benefit from a geometrically richer virtual specimen. In future work this would be interesting for further investigations. In order to clarify this, we added text in bold to the manuscript:*

*"Distortions are achieved by applying random rotations in the range of [-3°, 3°] and by scaling the object with a factor in the range of [0.9, 1.1]. Those values were chosen in order to sufficiently cover variations as seen in production, and in the data acquisition process assuming a robotic inspection system, such as manufacturing tolerances, calibration inaccuracies, and robot path inaccuracies."*

- The authors should explain the advantages offered by their ray-tracing solution. The description of the approach used for the simulation of sound propagation occupies most part of the manuscript. In my opinion, the authors should explain why existing approaches were not suitable to support their work on neural network training. In section 2.2, the authors state: “The presented approach is a simple and easy to implement simulation which does not take into account more complicated wave phenomena, which arise due to a directional dependent wave velocity.”. It is not clear why the authors have not tried to use existing and more accurate solutions. Was that deemed unnecessary? If that is the case, why?

*Thank you for the point raised. The scarcity of flawed training data is a general problem in the NDT field. This is even a common problem for training human inspectors. Machine learning requires even larger data sets, and the lack of representative flawed training data is a major challenge towards machine learning in the NDT field. Approaches towards training with synthetic data in current literature typically try to use accurate but very time-consuming FE simulations (most often, additionally, to a smaller set of real-world data). We added two recent and very interesting articles to our survey which speed up simulations by (i) mixing FE simulations (used in the defect locality) and ray-based simulations and by (ii) a deep learning approach. To clarify our point, we added the following to the paragraph “Ultrasonic image rendering” in chapter 1.1:*

*“However, wave-based simulations are typically very time-consuming, which makes it difficult to generate large data sets as required for deep learning. To reduce simulation time, in [Pyle2021] a mixture of finite element models in the defect locality and ray-based models for the whole region of interest is used. In [Gantala2021] a fast deep convolutional generative adversarial network is used to generate synthetic weld TFM images. For training, synthetic data is generated via finite element simulations.”*

*In the medical field, a lot of effort is put into developing methods for real-time rendering ultrasonic images (for training humans) based on a global illumination model. We think that the NDT field could benefit from these developments. Global illumination models are able to reproduce important image artefacts, as they incorporate indirect sound paths, but they typically do not incorporate low frequency effects. However, signal properties and image artefacts which are important for interpretation and utilized by human inspectors, such as wall-echo shadows and ghost artefacts, are reproduced with the presented simulation method. The hypothesis of this work is that with enough variability in simulation but reproducing import physical effects, the model will generalize to the real world (see chapter 2.2). The huge advantage of these global illumination models is that with a ray tracing approach the solution can be approximated very time efficient (medical sonography can be simulated in real-time). With the implemented simulator it is possible to generate a large data set within a short period of time, although the implementation is not optimized in any means (a single TFM image can be rendered in about a minute). Further, a major aim of our work is to investigate, if it is possible to achieve reasonable results using simple simulation models via domain randomization. Investigations of the influence of the accuracy of the used model on the detection accuracy would be interesting for future work. To clarify the advantage of the proposed ray-tracing solution, we added the following paragraph at the beginning of chapter 3:*

*“The introduced ray-tracing based simulator requires about one minute of computation time on a standard office computer for rendering a single TFM image. Although the implementation was not optimized towards speed efficiency, this allows to generate a large data set in reasonable period of time. Further, the simulator reproduces important physical effects and image artifacts which are used for data interpretation by human inspectors, i.e. reverberation, backwall attenuation, and reflections caused by delaminations. This makes the proposed method a good candidate for enabling domain randomized training.”*

*References:*

*[Pyle2021] Pyle, Richard J., et al.* *"Deep learning for ultrasonic crack characterization in NDE." IEEE Transactions on Ultrasonics, Ferroelectrics, and Frequency Control 68.5 (2020): 1854-1865.* *[Gantala2021] Gantala T., Balasubramaniam K. "Automated defect recognition for welds using simulation assisted TFM imaging with artificial intelligence." Journal of Nondestructive Evaluation 40.1 (2021): 1-24.*

- In the text following Equation 8, replace “refracted ray Θ _1_” with “refracted ray Θ _2_”.

*Thank you very much for pointing out that typographical error. We corrected the index in the manuscript.*

- In section 3, the following statement seems to contain a contradiction: “An interesting detail is that the method correctly classifies defects near the frontwall. Furthermore, the echo from a defect close to the top surface is correctly classified as background, i.e. as “no defect” (yellow arrow in Figure 8).”. I think the two sentences in the text above refer to the same subject, but have the opposite meaning.

*Unfortunately, that was not cleary phrased. The defect close to the top surface causes several echos, therefore, the defect is visible a second time in the TFM images. However, only the signal close to the top surface corresponds to the real defect. The second echo is a ghost artifact. Such ghost artefacts are typical in TFM images and are also produced by the developed simulator used in this work. We wanted to point out that the trained network correctly classifies this second echo as no defect. The text was changed to: "An interesting detail is that the method correctly classifies defects near the frontwall. Furthermore, the ghost artifact, which actually is just an echo of a real defect close to the top surface, is correctly classified as background, i.e. as “no defect” (yellow arrow in Figure 8).*
